# Case Report: Transthoracic echocardiographic diagnosis of agenesis of mitral chordae tendineae with papillary muscle fusion

**DOI:** 10.3389/fcvm.2026.1829935

**Published:** 2026-05-07

**Authors:** Xinyu Xue, Meiling Liu, Meiju Zhang, Yunlong Cao, Jianbo Wu, Qian Liu

**Affiliations:** 1 Department of Cardiology, Binzhou Medical University Hospital, Binzhou, Shandong, China; 2 Department of Rehabilitation Medicine, Binzhou Medical University Hospital, Binzhou, Shandong, China

**Keywords:** agenesis of mitral chordae tendineae, case report, congenital mitral valve malformation, echocardiography, papillary muscle fusion

## Abstract

The complete absence of mitral valve chordae tendineae together with fusion of the papillary muscles constitutes an exceedingly uncommon congenital anomaly, seldom encountered in clinical practice and prone to diagnostic oversight. We describe an adult patient in whom transthoracic echocardiography (TTE) provided definitive preoperative identification of the malformation, later corroborated by direct surgical inspection. The 50-year-old man presented with progressive exertional dyspnea and atrial fibrillation. Comprehensive TTE revealed redundant mitral leaflets inserting directly onto fused papillary muscles along the posterior left-ventricular wall, with total absence of the subvalvular chordal apparatus, resulting in severe mitral stenosis, moderate-to-severe regurgitation, secondary tricuspid regurgitation, and pulmonary hypertension. Mechanical mitral valve replacement and tricuspid annuloplasty yielded an uneventful recovery. This report underscores TTE's pivotal role in delineating the full mitral apparatus and refines the diagnostic framework for this rare entity.

## 1 Introduction

Congenital Mitral Valve Dysplasia encompasses a heterogeneous spectrum of structural anomalies resulting from embryonic developmental defects in the mitral valve apparatus (1). Although these lesions represent only 0.2–0.4% of congenital heart disease, their morphological heterogeneity frequently complicates both diagnosis and management (2). As critical fibrous structures, mitral chordae tendineae connect the valve leaflets to the papillary muscles and prevent blood regurgitation during ventricular systole by restricting excessive leaflet displacement (3, 4). Most chordal pathology is acquired—rupture or elongation from degeneration, infection, or rheumatic disease (5–7)—yet complete agenesis of the chordae combined with papillary muscle fusion remains exceptionally rare and is easily mistaken for more common acquired valvular disorders.

Here we present an adult case initially recognized by TTE and confirmed intraoperatively. The patient exhibited severe mixed mitral dysfunction without identifiable acquired causes such as rheumatic fever. By detailing the characteristic echocardiographic signature and systematically reviewing diagnostic differentials, this report aims to heighten clinical awareness of this malformation.

## 2 Case presentation

### 2.1 Clinical history

A 50-year-old male was admitted with exertional chest tightness and dyspnea ongoing for two months, which acutely deteriorated in the five days prior to admission. Symptoms were initially relieved by rest but later progressed to include paroxysmal nocturnal dyspnea, cough, and expectoration. The patient had received treatment at a local hospital prior to admission, including metoprolol tartrate for rate control of atrial fibrillation, with limited symptomatic improvement. Upon admission, he exhibited orthopnea and bilateral lower limb edema. The patient had no history of rheumatic fever, infective endocarditis, or familial cardiac disease.

### 2.2 Physical examination

Vitals**:** Temperature 36.6 °C, pulse 50 beats per minute, respiratory rate 15 breaths per minute, blood pressure 134/98 mmHg. Auscultation**:** Coarse bilateral breath sounds were noted without crackles or rhonchi. The first heart sound (S1) varied in intensity, with a distinct apical diastolic murmur. Extremities: Pitting edema was present in both lower extremities.

### 2.3 Ancillary investigations

**Figure 1:**
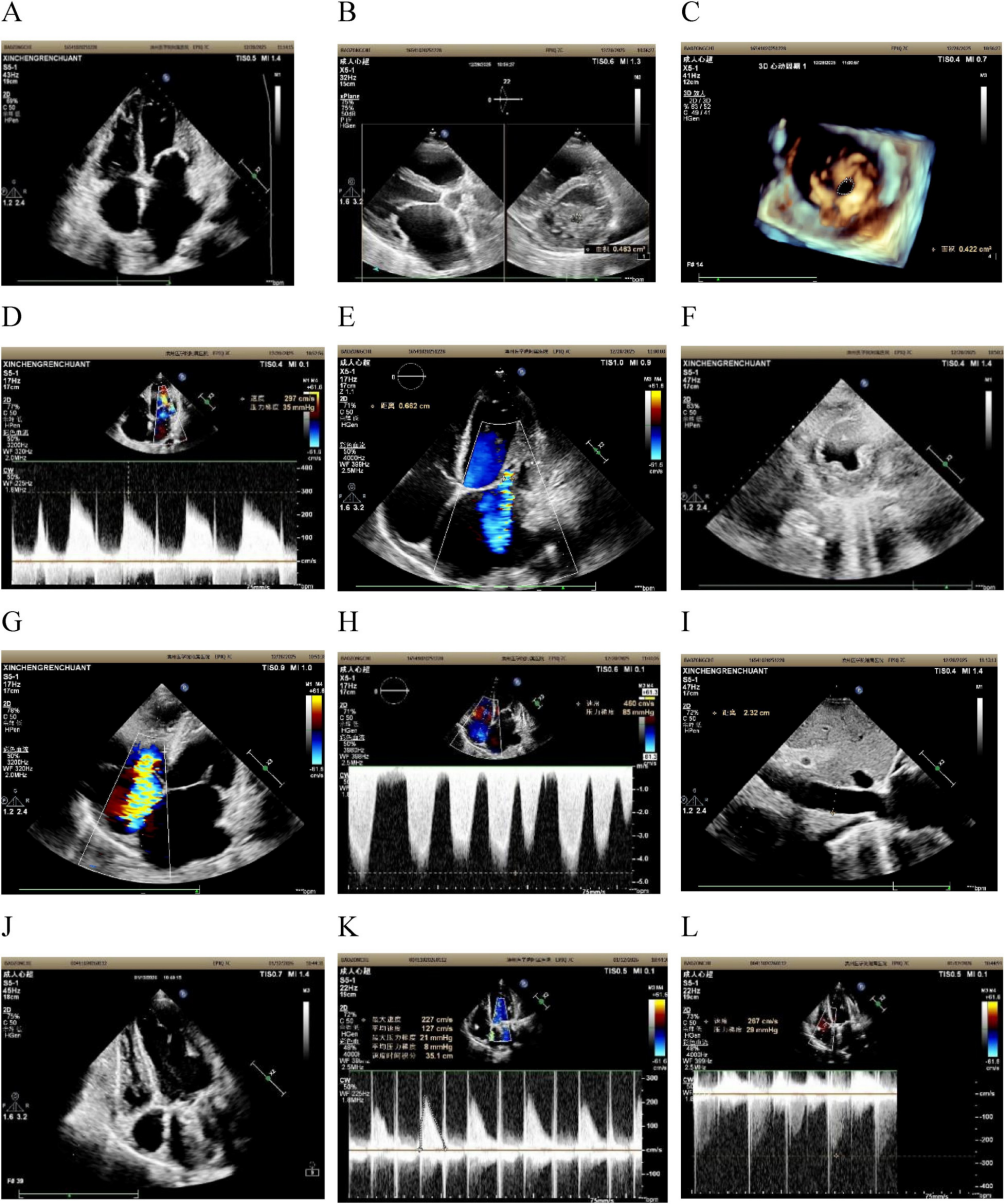
Preoperative echocardiographic findings of the patient **(A–I)**. **(A)** In the apical four-chamber view, redundant mitral valve leaflets are observed, with no definitive subvalvular chordae tendineae identified; the leaflets insert directly onto the papillary muscles. **(B)** Mitral valve orifice area (MVOA) is measured at approximately 0.463 cm^2^ via X-plane imaging. **(C)** Three-dimensional echocardiography (3DE) confirms mitral stenosis, with a planimetered mitral orifice area of approximately 0.422 cm^2^. **(D)** CWD demonstrates markedly elevated antegrade mitral flow, with a peak velocity of 297 cm/s and a corresponding peak transvalvular pressure gradient of 35 mmHg. **(E)** Moderate-to-severe mitral regurgitation is visualized in the apical four-chamber view, with vena contracta width of 6.6 mm. **(F)** Parasternal short-axis view at the papillary muscle level identifies two papillary muscle groups, which are confluent at the posterior aspect of the left ventricle. **(G)** The apical four-chamber view reveals severe tricuspid regurgitation. **(H)** CWD detects a peak tricuspid regurgitation velocity of 460 cm/s, yielding a peak transtricuspid pressure gradient of 85 mmHg. I: Subxiphoid view demonstrates a dilated inferior vena cava (IVC), with an end-diastolic internal diameter of 23 mm. Postoperative echocardiograms of the patient **(J–L)**. **(J)** Apical four-chamber view clearly visualizing the mechanical mitral valve prosthesis. **(K)** CWD interrogation demonstrates markedly reduced antegrade flow velocity across the mitral valve orifice relative to preoperative measurements. **(L)** CWD interrogation showing significantly diminished tricuspid regurgitant velocity compared with preoperative baseline assessments.

TTE demonstrated dysplasia of the mitral valve apparatus. The apical four-chamber view showed redundant, thickened mitral valve leaflets without chordae tendineae and no evidence of valvular or subvalvular calcification (Figure 1A). Severe mitral stenosis was seen on both xPlane (mitral valve area:0.46cm2) and three-dimensional echocardiography(3DE) (mitral valve area:0.42cm2) (Figures 1B,C). Continuous-Wave Doppler (CWD) measured the E peak: 2.97 m/s, transmitral gradient:35mmHg (Figure 1D) and vena contracta width: 6.6 mm (Figure 1E). Left atrial enlargement (anteroposterior diameter 64 mm), left-ventricular ejection fraction 43%, severe tricuspid regurgitation (Figure 1H), systolic pulmonary-artery pressure 85 mmHg, and inferior vena cava width:23 mm inspiratory collapse <50% (Figure 1I). Parasternal short-axis view at papillary muscle level showed fusion of the two papillary muscles in the posterior left ventricle (Figure 1F). 3DE provided an en-face “surgical” view of the narrowed orifice (Figure 1C). These findings collectively excluded acquired causes and pointed to a primary developmental defect (3).

### 2.4 Surgical intervention and postoperative course

**Figure 2:**
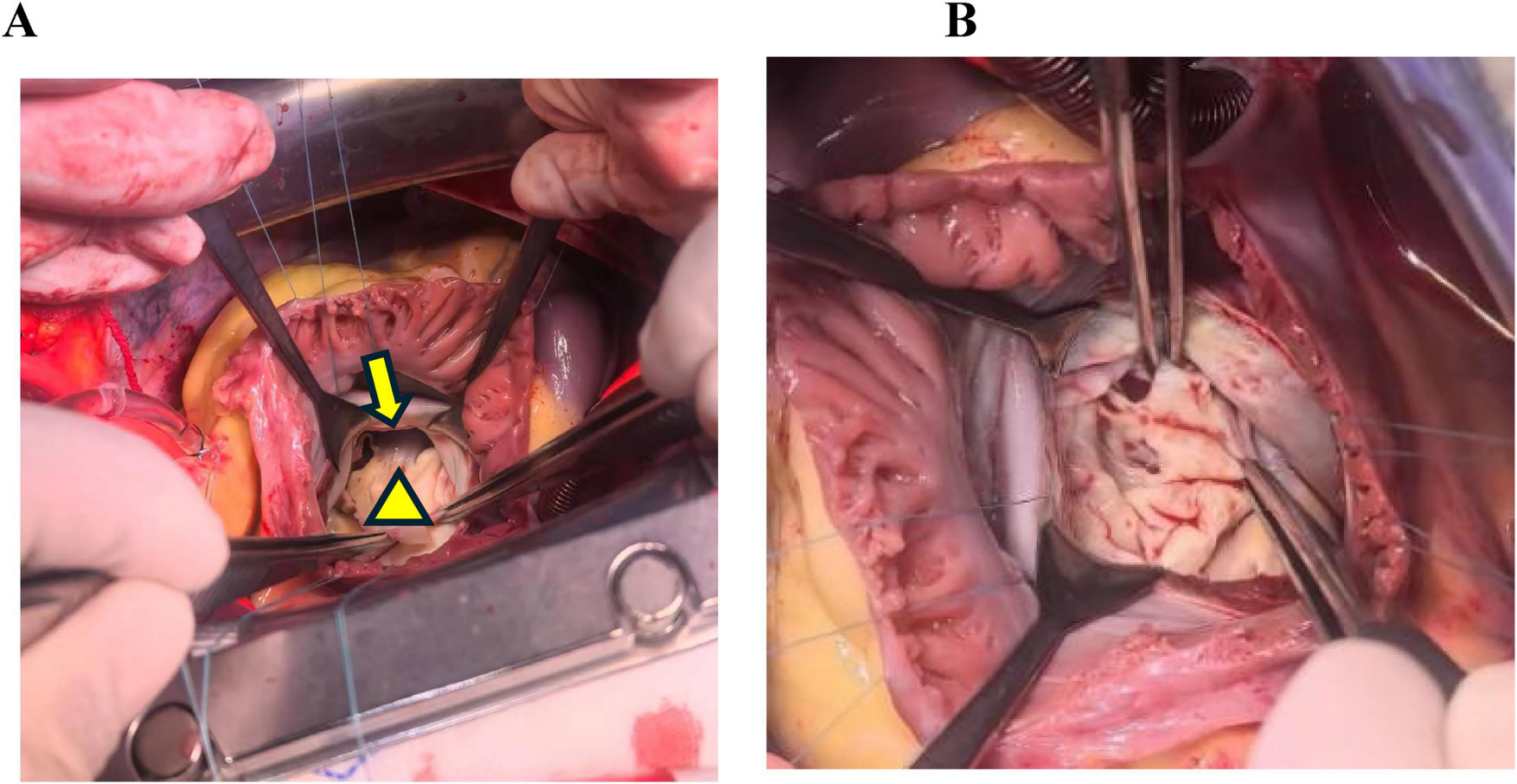
Intraoperative findings of the patient. **(A)** Following incision of the anterior mitral leaflet, the leaflet was found to insert directly onto the papillary muscles, with complete absence of chordae tendineae. **(B)** Intraoperative visualization of the stenotic mitral valve orifice. Triangle denotes the mitral valve leaflet; arrow indicates the papillary muscle.

**Figure 3:**
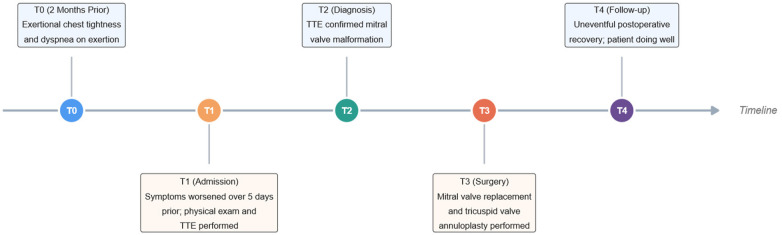
Clinical timeline of the patient's presentation, diagnosis, and management.

During the same admission, one week after the final diagnosis, intraoperative findings were consistent with the preoperative TTE. Intraoperative TEE was performed and further corroborated the abnormal attachment of the mitral valve leaflets to the papillary muscles, along with associated hemodynamic changes (Supplementary Movies 1 and 2). Subsequent surgical inspection demonstrated these anatomical anomalies: the mitral valve leaflets were redundant and thickened, and chordae tendineae were absent, inserting directly into the fused papillary muscles (Figure 2). Subsequently, the mitral valve apparatus was resected and replaced with a 31 mm St. Jude Medical mechanical valve. Meanwhile De Vega tricuspid annuloplasty was performed to correct annular dilatation and severe regurgitation. The patient was uneventfully weaned from cardiopulmonary bypass (CPB), with sustained postoperative hemodynamic stability. Postoperative TTE (Figures 1J–L) showed suitable prosthetic valve position, lower transvalvular velocity, attenuated tricuspid regurgitation, and reduced pulmonary artery pressure. The clinical course is summarized in (Figure 3).

## 3 Discussion

### 3.1 Pathogenesis and clinical rarity

Complete absence of the mitral chordae tendineae with concomitant papillary muscle fusion is an extremely rare congenital cardiac malformation, for which clinical reports are exceedingly scarce, and the specific pathogenesis remains incompletely elucidated. Existing studies have hypothesized that this condition may be linked to impaired differentiation and remodeling of the mitral valve complex (encompassing the valve leaflets, chordae tendineae, and papillary muscles) during embryonic development (8). Owing to its extremely low incidence and non-specific imaging features, this condition is highly prone to missed diagnosis and misdiagnosis in clinical settings. The present case, preliminarily diagnosed via TTE and intraoperatively confirmed, offers valuable experience for the imaging identification and clinical decision-making of this rare disorder.

### 3.2 Anatomical classification within the Undifferentiated Chordae Mitral Valve Spectrum

From the perspective of embryonic development and anatomical classification, this case can be classified as a severe phenotype within the Undifferentiated Chordae Mitral Valve Spectrum. This spectrum describes a group of congenital malformations caused by impaired differentiation of chordae tendineae during the embryonic period, including Mitral Arcade, Hammock Mitral Valve, and Parachute Mitral Valve, among others (9). In this case, the mitral valve leaflets were directly connected to the papillary muscles with complete absence of chordae tendineae structures, consistent with the core defining features of this spectrum. However, this case also exhibited unique complexity: it simultaneously presented partial features of asymmetric parachute-like mitral valve (convergence of the subvalvular apparatus), but unlike classic parachute mitral valve (characterized by a single papillary muscle group), two papillary muscle groups were identified in this case, albeit fused at the posterior left ventricular wall. Likewise, it was distinct from classic Mitral Arcade, which typically features fibrous cords connecting two separate papillary muscles to the valve leaflets (10, 11). A case of congenital absence of mitral chordae tendineae reported in 2012 shared similarities with the present case, manifesting as direct adhesion of the valve leaflets to the papillary muscles (8). However, the additional anatomical variation of papillary muscle fusion in this case drives more complex pathological alterations and may represent an intermediate or mixed morphological variant across established classification subtypes.

### 3.3 Diagnostic contributions of transthoracic echocardiography

This case further corroborates the central role of TTE in the diagnosis of complex congenital mitral valve malformations. The rarity and complexity of this anomaly often lead to significant diagnostic challenges. In this context, multi-modal echocardiography serves as a bridge between clinical suspicion and surgical reality. While 2D TTE can clearly display morphological details including leaflet redundancy, chordae tendineae absence, and papillary muscle fusion, it may occasionally provide an incomplete assessment of spatial relationships. Compared with conventional 2D imaging, 3DE offers a more comprehensive view of these spatial relationships. When chordae tendineae abnormalities are suspected, three-dimensional echocardiography can provide high-resolution anatomical images from a “surgical view,” which facilitates definitive confirmation of the diagnosis and guides precise surgical planning (3, 9, 12). Furthermore, TTE allows for the accurate quantification of the severity of stenosis and regurgitation, as well as secondary hemodynamic changes (such as atrial enlargement and pulmonary hypertension) through color and spectral Doppler (3, 13–16). For such intricate lesions, echocardiographers should maintain a high level of vigilance and perform systematic multi-plane scanning, especially in the left ventricular short-axis view, which is critical for evaluating the morphology and location of the subvalvular apparatus (3, 9, 12).

### 3.4 Surgical management and therapeutic considerations

For congenital mitral valve malformation complicated with severe hemodynamic compromise, surgical intervention is the only curative treatment. Although mitral valve repair is the optimal strategy, the specific surgical procedure depends on the complexity of valvular lesions (17–20). For chordae tendineae agenesis, artificial chordae (e.g., expanded polytetrafluoroethylene, ePTFE) can be used to reconstruct the connection between valve leaflets and papillary muscles. However, mitral valve replacement becomes an indispensable option in the setting of intrinsic leaflet dysplasia with poor mobility, severe fusion of the subvalvular apparatus, and absence of a reparable basis (17, 21). In the present case, given the severe subvalvular stenosis caused by direct attachment of valve leaflets to fused papillary muscles, intrinsic leaflet redundancy with rigid mobility, and significant cardiac remodeling secondary to long-standing pathological changes, we determined that conditions for successful and durable repair were not met after comprehensive evaluation, and therefore performed mitral valve replacement with preservation of the posterior leaflet and partial subvalvular apparatus, along with concomitant management of tricuspid regurgitation. The favorable short-term postoperative outcomes have verified the efficacy of this therapeutic strategy. However, this patient requires lifelong anticoagulation monitoring, and the long-term prognosis remains to be further evaluated via systematic follow-up.

The primary strength of this case report is the clinical application of multi-modal echocardiography (2D and 3DE). These non-invasive techniques provided high-resolution morphological details, enabling an accurate preoperative diagnosis of the agenesis of mitral chordae tendineae and papillary muscle fusion, which was subsequently confirmed by surgical inspection. This case highlights that 3DE is an invaluable tool for characterizing complex subvalvular apparatus malformations.

As a single case report, this study has inherent limitations, and generalizable conclusions cannot be drawn. Histopathological analysis of the excised valve was not performed, which limited further microscopic characterization. In addition, although the immediate surgical outcome was favorable, longer-term follow-up is needed to assess prosthetic valve function, right heart remodeling, and the patient's quality of life.

This case serves as a critical reminder that congenital mitral valve anomalies can remain clinically silent until adulthood, with symptoms manifesting only after prolonged hemodynamic decompensation. Such cases may be easily misdiagnosed as acquired valvular diseases, such as rheumatic heart disease. Clinicians and sonographers should maintain a high clinical suspicion when encountering atypical subvalvular structures. A systematic echocardiographic evaluation focusing on the chordae and papillary muscle attachment is crucial for avoiding diagnostic oversight and ensuring precise surgical planning.

## 4 Conclusion

Agenesis of the mitral chordae tendineae with papillary muscle fusion is a rare congenital malformation capable of producing profound hemodynamic compromise even in adulthood. This case report highlights the value of transthoracic echocardiography as a first-line diagnostic tool, which, via meticulous and systematic scanning, can accurately identify the characteristic anatomical abnormalities of the leaflet-chordae tendineae-papillary muscle complex and provide critical information for clinical decision-making. Surgical intervention is an effective treatment modality, and mitral valve replacement can achieve favorable early outcomes when the lesions are complex and not amenable to repair. Enhanced recognition of this condition helps reduce misdiagnosis and improve patient prognosis.

## Patient perspective

For months I struggled to catch my breath and could not sleep flat. I had no idea the problem went back to how my heart developed. After the surgery the difference was immediate — I could breathe again, and I finally felt like myself.

## Data Availability

The original contributions presented in the study are included in the article/Supplementary Material, further inquiries can be directed to the corresponding author.
